# Large language model use in dental education: a cross-sectional multi-country study

**DOI:** 10.1080/10872981.2026.2707729

**Published:** 2026-08-06

**Authors:** Abubaker Qutieshat, Lovely M. Annamma, Gurdeep Singh, Mohd Hafiz Arzmi, Wan Nurhazirah Wan Ahmad Kamil, Lina Khasawneh, Sudhir Rama Varma, Jair Carneiro Leão, Biji Thomas George, Mohammad S. Alrashdan

**Affiliations:** a Restorative Dentistry, College of Dental Medicine, University of Sharjah, Sharjah, UAE; b Restorative Dentistry, Oman Dental College, Muscat, Oman; c Dental Research Cell, Dr. D. Y. Patil Dental College & Hospital, Dr. D. Y. Patil Vidyapeeth, Pune, Maharashtra, India; d Prosthodontics, College of Dentistry, Ajman University, Ajman, UAE; e Fundamental Dental and Medical Sciences, International Islamic University Malaysia, Pahang, Malaysia; f Melbourne Dental School, University of Melbourne, Victoria, Australia; g Special Care Dentistry Unit, Faculty of Dentistry, Universiti Teknologi MARA, Sungai Buloh Campus, Selangor, Malaysia; h Prosthodontics, Jordan University of Science and Technology, Irbid, Jordan; i Clinical Sciences, College of Dentistry, Ajman University, Ajman, UAE; j Oral Medicine, Federal University of Pernambuco (UFPE), Recife, Brazil; k Surgery, RAK College of Medical Sciences, RAK Medical and Health Sciences University, Ras Al Khaimah, UAE; l Oral and Craniofacial Health Sciences, College of Dental Medicine, University of Sharjah, UAE

**Keywords:** Academic Integrity, Artificial Intelligence, ChatGPT, dental education, large language models

## Abstract

**Background:**

Large language models (LLMs) are increasingly used in higher education, but multi-country evidence on dental students’ use, verification, and integrity practices is limited.

**Objective:**

To compare senior dental students’ LLM use, perceived time and academic impact, reliability judgements, verification practices, and integrity safeguards across five countries.

**Methods:**

An anonymous cross-sectional online survey was administered to final-year dental students in the United Arab Emirates (UAE), Jordan, Malaysia, Oman, and Brazil. Measures included tools used, frequency and motivations, learning activities, perceived time and academic impact, verification frequency and strategies, guideline awareness, and integrity safeguards. Analyses used Kruskal-Wallis and chi-square tests with Benjamini-Hochberg adjustment, effect sizes, Spearman correlations, and ordinal logistic models.

**Results:**

In total, 454 students participated (UAE 160, Jordan 101, Malaysia 75, Oman 62, Brazil 56; mean age 22.9; 74.9% female). ChatGPT predominated (95.9%), followed by Gemini, formerly Bard (18.0%), DeepSeek (16.4%), and Claude (7.4%). Tool diversity varied across country-based cohorts, with Oman showing greater multi-tool uptake. Use was frequent (several times/week 39.2%, daily 28.6%). Key motivations were saving time (73.0%), clarifying concepts (56.9%), and summarising (54.1%). Common activities included understanding complex concepts (75.3%), summarising lecture notes (70.0%), exam preparation (61.5%), and assignment research (53.2%); exam-time assistance was reported by 25.6%. Verification was ‘always’ 20.0% and ‘often’ 34.1%, varying across country-based cohorts, with Oman verifying less frequently than other cohorts. Guideline awareness was 40.3% overall (UAE 61.3% vs Brazil 8.3%). Integrity safeguards commonly involved paraphrasing (69.6%), citations (39.2%), and plagiarism checks (38.0%); disclaimers were uncommon (9.2%). LLM-use frequency correlated with broader academic use (ρ = 0.289) but not with integrity concern (OR = 0.963).

**Conclusions:**

LLM use is widespread and heterogeneous across settings, including non-trivial higher-stakes use. Dental programmes should implement explicit training in verification, evidence traceability, and disclosure, supported by clear, enforceable guidance and assessment designs aligned with real-world LLM practices.

## Introduction

Large language models (LLMs) have rapidly permeated higher education, offering new possibilities for academic writing, exam preparation, and personalised learning [1–3]. In dental education, where evidence-based practice and critical thinking are paramount, the integration of LLMs presents both opportunities and challenges [4]. Dental students are increasingly adopting LLMs for academic tasks, but their usage patterns, perceived academic outcomes, verification behaviours, and risk perceptions appear to be shaped by a complex interaction of ethical concerns and institutional guidance [3–8].

Early scholarly and policy-oriented discussion around LLMs has understandably centred on assessment integrity, academic misconduct and potential risks to critical thinking and independent judgement [4,6,9]. However, multiple reviews and surveys indicate that empirical evidence is still developing, uneven across contexts and often dominated by cross-sectional, single-setting or single-country samples, which limits what can be inferred about generalisable versus context-specific patterns [2,10]. In parallel, analyses of institutional AI documents suggest that institutional responses have frequently prioritised academic honesty language over more comprehensive implementation and review mechanisms, contributing to variability in what students are told and how use is governed [6–9].

Moreover, educational consequences of LLM use are unlikely to be binary. Students can report strong perceived usefulness and efficiency while simultaneously acknowledging ethical concerns and reliability risks, particularly in assessment-related contexts [9]. Evidence from medical and dental education suggests that responsible use requires more than measuring frequency of use [4,11,12]. It also requires attention to students’ purposes for using LLMs, perceived benefits, and verification routines for managing hallucinations, fabricated citations, and plausible-sounding misinformation. This also implies that understanding student practice requires examining how LLM outputs are evaluated against conventional academic resources and credible evidence, rather than treating LLMs as standalone answer tools [4,6].

In dentistry and medical education, where clinical reasoning, up-to-date knowledge, and autonomous judgement are core outcomes, these distinctions become especially consequential [4]. Reviews in dental education highlight discipline-specific risks, including inaccuracies, false citations, and limitations in image-dependent learning tasks [13]. These risks strengthen the case for clearer guidance and explicit training in verification and critical evaluation. At the same time, institutional policy analyses underline that governance maturity, transparency expectations, and practical implementation guidance remain variable, which may partly explain heterogeneity in student behaviours and perceptions across settings [6–9].

This study therefore aimed to examine cross-country differences in dental students’ LLM use, perceived time and academic impact, reliability judgements, verification practices, and integrity-related safeguards.

## Materials and methods

A cross-sectional questionnaire-based study was conducted using an anonymous online survey administered to senior undergraduate dental students across five countries: the United Arab Emirates (UAE), Jordan, Malaysia, Oman, and Brazil. Participating institutions were identified through the research team’s academic networks. The participating institutions were therefore treated analytically as country-based institutional cohorts rather than nationally representative samples. The survey was disseminated across six dental institutions, with two institutions in the UAE and one institution in each of Jordan, Malaysia, Oman, and Brazil. The survey link was shared through local institutional channels. Participation was voluntary and no identifying information was collected.

Eligible participants were students enroled in final-year dental programmes in the participating countries. The instrument comprised demographic and background items (age, gender, perceived AI proficiency based on self-rating, and familiarity with the concept of large language models), followed by sections assessing: LLM tools used, frequency of use, motivations for use, academic activities supported by LLMs, perceived time impact, ease of learning to use LLMs, and perceived academic outcome impact, comparative judgements of LLM accuracy relative to conventional resources (textbooks/lectures, peer-reviewed articles, and online databases/forums), verification frequency and verification strategies, awareness of institutional or departmental AI guidelines and perceived influence on usage, integrity safeguards used to avoid plagiarism or maintain academic integrity, and perceived impact on critical thinking. The full survey structure, item mapping, response options, and analysis coding are provided in Supplementary file S1.

Ethical approval for the overall study protocol was obtained from the Research Ethics Committee of the University of Sharjah (Ref REC-25-04-06-02-F). The study was conducted in accordance with the principles of the Declaration of Helsinki. Before accessing the survey, participants viewed an information sheet and provided electronic informed consent by selecting an ‘I agree’ option. The survey was anonymous, participation was voluntary, and respondents could exit the survey at any time without penalty.

Analyses were conducted in R (version 4.4.3; R Foundation for Statistical Computing). Categorical variables are reported as *n* (%) and continuous/ordinal variables as mean ± SD and median (IQR). Cross-country differences in single-choice ordinal items were tested using Kruskal-Wallis tests with Dunn post hoc comparisons when applicable, applying Benjamini-Hochberg (BH) adjustment for multiple testing. For multiple-selection questions, each option was analysed as a binary outcome (selected vs not selected) using chi-square tests with BH adjustment within each item set. Effect sizes were summarised using η^2^H for Kruskal-Wallis tests and Cramer’s V for categorical comparisons. Associations between LLM-use frequency and derived breadth indices were assessed using Spearman correlations with BH correction. Key ordinal outcomes (verification frequency and integrity concern) were further examined using cumulative link (ordinal logistic) models including country and LLM-use frequency. Proportional-odds adequacy was assessed, and model-based predicted probabilities were generated for interpretation. Regression results are presented as odds ratios (OR) with 95% confidence intervals, all tests were two-sided. Sensitivity analyses excluding the smallest country cohort yielded materially similar effect directions and inferences for the key ordinal regression and correlation findings.

## Results

### Participant characteristics and overall LLM use

A total of 454 respondents were analysed across five countries: UAE (*n* = 160), Jordan (*n* = 101), Malaysia (*n* = 75), Oman (*n* = 62), and Brazil (*n* = 56). The mean age was 22.9 ± 2.5 years (median 23, IQR 21–24, range 20–34). Female respondents comprised 74.9% of the sample. Perceived AI proficiency, based on self-rating, was predominantly intermediate (63.1%), followed by beginner (20.7%), advanced (14.1%), and expert (2.1%). Overall familiarity with LLMs as a concept and tool was high (94.2%).

ChatGPT was the most widely used LLM (95.9%), followed by Gemini, formerly Bard (18.0%), DeepSeek (16.4%), and Claude (7.4%). All other tools were each used by fewer than 2% of respondents. ChatGPT use was near universal in the UAE (100%), Jordan (97.0%), and Malaysia (97.3%), with slightly lower uptake in Brazil (91.7%) and Oman (83.9%). Secondary-tool use varied across countries. Oman showed the strongest tendency towards multi-tool use, with the highest uptake of Gemini, formerly Bard (41.9%) and Claude (24.2%), whereas the UAE showed higher uptake of DeepSeek (18.1%) than Gemini, formerly Bard (7.5%).

LLM use was frequent overall. The most common pattern was use several times a week (39.2%), followed by daily or almost daily use (28.6%) and weekly use (16.1%). Monthly use (7.6%), rare use (7.4%), and no use (1.2%) were less common.

### Motivations and academic use

The most commonly reported motivations and academic uses of LLMs are summarised in Table 1. Overall, students most frequently used LLMs for time saving, conceptual clarification, summarisation, exam preparation, and assignment-related research. Higher-stakes uses were less common but still present, including examination assistance and generating or draughting academic papers.

**Table 1.** Reported motivations and academic uses of LLMs.

Category	Item	Percentage of respondents
Motivation for using LLMs	Saving time	73.0%
	Improving understanding of difficult concepts	56.9%
	Generating quick summaries	54.1%
Academic activity	Understanding complex concepts	75.3%
	Summarising lecture notes	70.0%
	Exam preparation	61.5%
	Assignment-related research	53.2%
	Clarifying lecture content	51.2%
	Generating or writing assignments	38.2%
Higher-stakes academic use	Generating academic papers	25.8%
	Using LLMs to help solve problems during examinations	25.6%
	Draughting research papers	24.5%

Note: Multiple responses were permitted. Percentages refer to the proportion of respondents selecting each item.

The largest cross-country difference was observed for using LLMs to understand complex medical or dental concepts, with high endorsement in Malaysia (93.3%), the UAE (87.5%), and Jordan (83.2%), but lower endorsement in Brazil (38.9%) and Oman (30.6%). Exam or quiz preparation was highest in the UAE (76.2%) and Malaysia (68.0%) and lowest in Oman (32.3%). Assignment-related research was also more commonly reported in the UAE (65.0%) and Malaysia (62.7%) than in Oman (29.0%).

**Figure 1.**
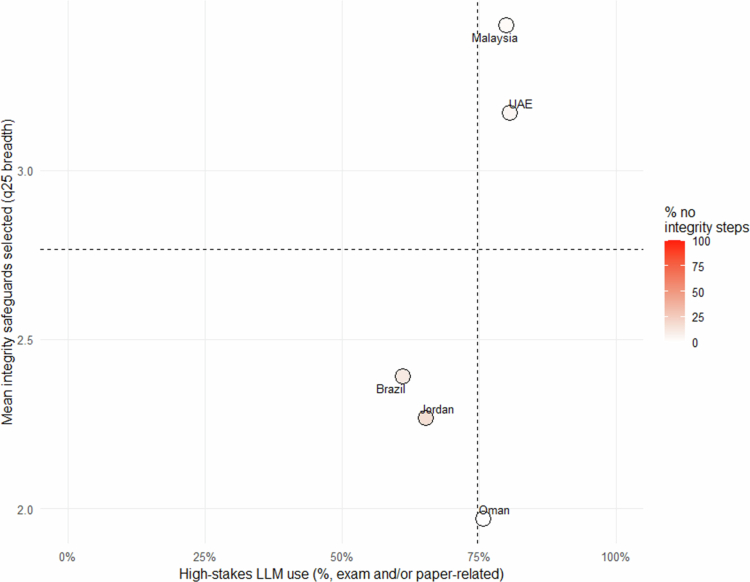
Country-level relationship between high-stakes LLM use and integrity safeguards. The x-axis shows the percentage of respondents endorsing at least one high-stakes academic use (exam or examination-related assistance and/or draughting research or academic papers). The y-axis shows the mean number of integrity safeguards selected. Point fill represents the percentage reporting no integrity steps (0 = white, 100 = red). Dashed lines indicate overall sample means.

Higher-stakes academic uses also varied across countries. Examination assistance was most frequently endorsed in Oman (37.1%) and Malaysia (34.7%) and least frequently in Brazil (11.1%) and the UAE (16.9%). Country-level alignment between higher-stakes LLM uses and integrity safeguards is illustrated in Figure 1.

### Perceived impact and comparative judgements

Regarding perceived time impact, 63.6% reported saving more time overall, 16.8% reported no change, and 19.6% reported spending more time overall. This differed across countries, with Oman showing a shift towards greater time investment relative to the other cohorts.

Learning to use LLMs effectively for academic tasks was generally perceived as easy or moderately easy. Overall, 54.6% described this as very or somewhat easy, 30.0% as moderate, and 15.4% as somewhat or very challenging. Country differences were observed, with UAE respondents reporting the lowest difficulty and Oman respondents reporting the highest difficulty.

Most respondents perceived a positive academic impact from LLM use. Overall, 55.8% reported that LLMs improved their academic outcomes, 25.1% reported no change, and 19.1% were not sure. No respondents reported worsening academic outcomes.

Perceived effects on critical thinking differed across cohorts. Responses suggested a more negative perceived impact in the UAE, Jordan, Malaysia, and Brazil, whereas Oman showed a more neutral profile. Most respondents also agreed that LLMs help keep learning up to date (62.0%), although Oman was more sceptical than the UAE, Jordan, and Malaysia, while Brazil showed an intermediate pattern. Comparative judgements of LLM outputs against textbooks or lectures, peer-reviewed articles, and online sources also differed across countries, although the effect sizes were generally small. The comparison against exam manuals did not differ significantly, and the question-bank comparison item could not be robustly compared across all countries due to sparse cells.

### Verification practices

Verification behaviour showed a mixed profile. Overall, 20.0% of respondents reported always verifying LLM response accuracy, 34.1% often, 25.3% sometimes, 17.3% rarely, and 3.2% never. Verification frequency differed across countries. Oman reported lower verification frequency than the other cohorts, whereas UAE respondents reported relatively frequent verification.

**Figure 2.**
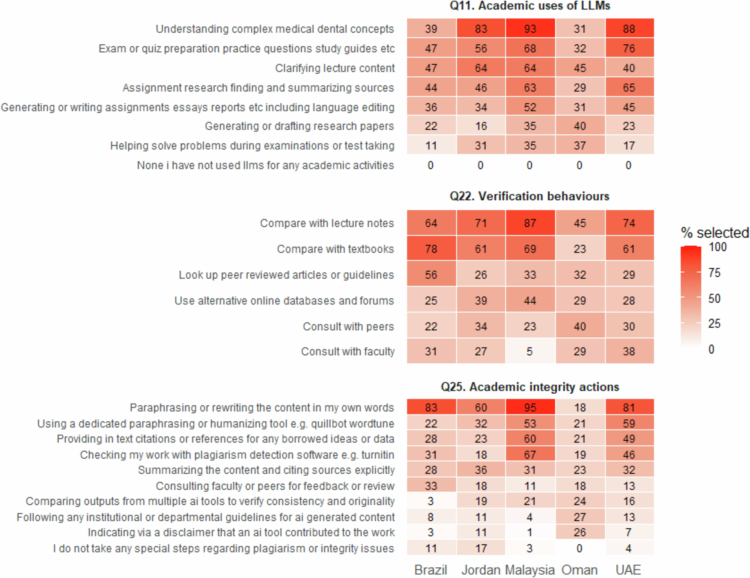
Country-level heat map of LLM-related behaviours and integrity practices. Tiles show the percentage of respondents within each country who selected each item (academic uses of LLMs, verification behaviours, and academic integrity actions). Values are displayed as percentages within tiles, colour intensity ranges from white (0%) to red (100%), with darker tiles indicating higher endorsement.

Verification strategies also varied across countries. Cross-checking outputs against textbooks and lecture notes differed notably, with Oman reporting lower use of these strategies than the other cohorts. Consulting faculty also varied, ranging from 37.5% in the UAE to 5.3% in Malaysia. Looking up peer-reviewed articles or guidelines showed smaller but significant variation, with Brazil reporting the highest uptake. Consulting peers did not differ significantly after correction. Country-level patterns across academic uses, verification behaviours, and integrity practices are summarised in Figure 2.

### Institutional guidance and integrity safeguards

Overall, 40.3% of respondents reported awareness of institutional or departmental guidelines on AI use, 31.3% were not sure, and 28.3% reported no awareness. Awareness differed across countries, being highest in the UAE (61.3%) and lowest in Brazil (8.3%). Among respondents who answered the follow-up question on guideline impact (*n* = 176), 51.7% reported that guidelines influenced their usage somewhat and 40.3% reported that they influenced their usage significantly.

Most respondents reported using at least one integrity-related safeguard when working with LLM-generated content. The most common practice was paraphrasing or rewriting in one’s own words (69.6%), followed by using paraphrasing or humanising tools (43.1%), providing in-text citations or references (39.2%), using plagiarism-detection software (38.0%), and summarising while explicitly citing sources (31.1%). Less frequent practices included cross-checking outputs across multiple AI tools (17.7%), consulting faculty or peers for review (16.1%), following institutional or departmental guidelines (12.7%), and disclosing AI contribution through a disclaimer (9.2%). Overall, 6.9% reported taking no special steps.

Integrity safeguards varied substantially across countries. Paraphrasing or rewriting was common in Malaysia (94.7%) and the UAE (80.6%) but less common in Oman (17.7%). Plagiarism-detection use was highest in Malaysia (66.7%) and the UAE (46.2%) and lower in Jordan (17.8%) and Oman (19.4%). Providing in-text citations was highest in Malaysia (60.0%) and lowest in Oman (21.0%). Disclosure through a disclaimer and reported adherence to institutional guidelines were comparatively most frequent in Oman, whereas taking no special steps was most frequent in Jordan.

Concern about plagiarism or academic integrity issues arising from LLM use differed across cohorts. Median concern was higher in the UAE, Jordan, Malaysia, and Brazil than in Oman. In an ordinal regression model including country and LLM-use frequency, use frequency was not associated with integrity concern. Compared with the UAE, Jordan and Oman showed lower odds of reporting higher concern.

### Associations with LLM-use frequency

Across the full sample, higher LLM-use frequency was associated with broader use of LLMs across academic activities. A smaller positive association was observed between use frequency and integrity-safeguard breadth. However, use frequency was not significantly associated with verification frequency, verification breadth, perceived plagiarism concern, or perceived effects on critical thinking after correction.

**Figure 3.**
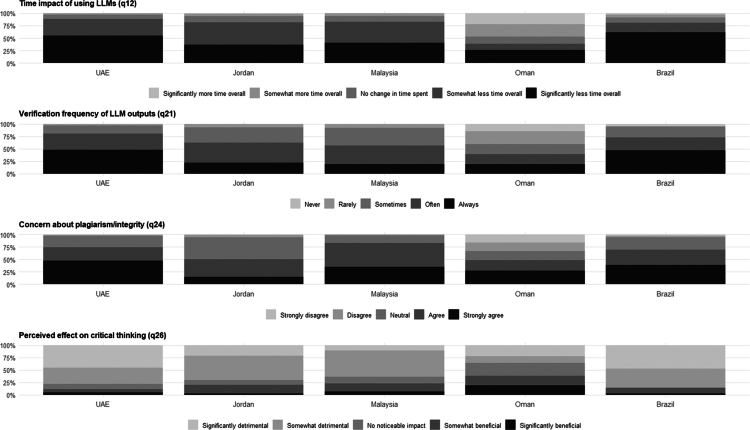
Distribution of responses to Likert-scale items by country. Bars show the percentage of respondents within each country selecting each response category (100% stacked bars). Shading indicates response level from lighter to darker grey (lower to higher endorsement). Percentages are based on non-missing responses.

Country-stratified analyses showed that the association between use frequency and academic-activity breadth was present in Jordan, the UAE, Brazil, and Malaysia, but not in Oman. In Malaysia, use frequency was negatively associated with verification breadth. Country-wise response distributions for Likert-scale items are shown in Figure 3. A summary of the key inferential analyses, including statistical tests, *p*-values, effect sizes, and main interpretations, is provided in Table 2.

**Table 2.** Summary of key inferential analyses.

Analysis	Test	*p*-value/adjusted p	Effect size	Main finding
LLM tools: Tool diversity by country	Kruskal-Wallis	*p* < 0.001	η^2^H = 0.047	Tool diversity differed across countries, with Oman showing greater multi-tool uptake.
Academic activities: Understanding complex medical/dental concepts	Chi-square with BH adjustment	adjusted *p* < 0.001	V = 0.529	Highest in Malaysia, UAE, and Jordan; lower in Brazil and Oman.
Academic activities: Exam or quiz preparation	Chi-square with BH adjustment	adjusted *p* < 0.001	V = 0.313	Highest in UAE and Malaysia; lowest in Oman.
Academic activities: Assignment-related research	Chi-square with BH adjustment	adjusted *p* < 0.001	V = 0.262	Higher in UAE and Malaysia than Oman.
Higher-stakes use: Use during examinations	Chi-square with BH adjustment	adjusted *p* < 0.001	V = 0.211	Highest in Oman and Malaysia; lowest in Brazil and UAE.
Higher-stakes use: Generating or draughting research papers	Chi-square with BH adjustment	adjusted *p* = 0.003	V = 0.192	Significant cross-country variation.
Perceived impact: Time impact	Kruskal-Wallis	*p* < 0.001	η^2^H = 0.109	Oman shifted towards greater time investment.
Usability: Difficulty learning to use LLMs	Kruskal-Wallis	*p* < 0.001	η^2^H = 0.169	UAE reported lower difficulty; Oman reported higher difficulty.
Academic outcomes: Perceived academic outcome impact	Pearson χ^2^ with Monte Carlo simulation	*p* = 0.00005	V = 0.267	Perceived academic impact differed across cohorts.
Critical thinking: Perceived effect on critical thinking	Kruskal-Wallis	*p* < 0.001	η^2^H = 0.099	Oman showed a more neutral profile than several other cohorts.
Learning currency: Belief that LLMs keep learning up to date	Kruskal-Wallis	*p* < 0.001	η^2^H = 0.185	Oman was more sceptical than UAE, Jordan, and Malaysia.
Resource comparison: LLMs versus textbooks/lectures	Kruskal-Wallis	*p* < 0.001	η^2^H = 0.040	Significant but small cross-country differences.
Resource comparison: LLMs versus peer-reviewed articles	Kruskal-Wallis	*p* < 0.001	η^2^H = 0.045	Significant but small cross-country differences.
Resource comparison: LLMs versus online sources/forums	Kruskal-Wallis	*p* = 0.002	η^2^H = 0.029	Significant but small cross-country differences.
Resource comparison: LLMs versus exam manuals	Kruskal-Wallis	*p* = 0.716	η^2^H negligible	No significant cross-country difference.
Verification: Verification frequency	Kruskal-Wallis	*p* < 0.001	η^2^H = 0.119	Oman reported lower verification frequency than other cohorts.
Verification strategies: Comparing outputs with textbooks	Chi-square with BH adjustment	adjusted *p* < 0.001	V = 0.315	Oman reported lower use of textbook cross-checking.
Verification strategies: Comparing outputs with lecture notes	Chi-square with BH adjustment	adjusted *p* < 0.001	V = 0.264	Oman reported lower use of lecture-note cross-checking.
Verification strategies: Consulting faculty	Chi-square with BH adjustment	adjusted *p* < 0.001	V = 0.248	Use varied across countries, highest in UAE and lowest in Malaysia.
Verification strategies: Looking up peer-reviewed articles/guidelines	Chi-square with BH adjustment	adjusted *p* = 0.031	V = 0.163	Brazil reported the highest uptake.
Verification strategies: Consulting peers	Chi-square with BH adjustment	adjusted *p* = 0.155	V = 0.124	No significant difference after correction.
Guidelines: Awareness of institutional/departmental AI guidelines	Chi-square	*p* < 0.001	V = 0.316	Awareness was highest in UAE and lowest in Brazil.
Integrity safeguards: Paraphrasing or rewriting	Chi-square with BH adjustment	adjusted *p* < 0.001	V = 0.520	Common in Malaysia and UAE; less common in Oman.
Integrity safeguards: Plagiarism-detection use	Chi-square with BH adjustment	adjusted *p* < 0.001	V = 0.366	Highest in Malaysia and UAE; lower in Jordan and Oman.
Integrity safeguards: Providing in-text citations/references	Chi-square with BH adjustment	adjusted *p* < 0.001	V = 0.313	Highest in Malaysia and lowest in Oman.
Integrity safeguards: Disclosure via disclaimer	Chi-square with BH adjustment	adjusted *p* < 0.0001	V = 0.259	Most frequent in Oman.
Integrity safeguards: Following institutional/departmental guidelines	Chi-square with BH adjustment	adjusted *p* = 0.003	V = 0.205	Most frequent in Oman.
Integrity safeguards: Consulting faculty or peers for review	Chi-square with BH adjustment	adjusted *p* = 0.034	V = 0.159	Highest in Brazil.
Integrity concern: Concern about plagiarism or academic integrity	Kruskal-Wallis	*p* < 0.001	η^2^H = 0.091	Concern was lower in Oman than in the other cohorts.
Ordinal model: Integrity concern predicted by LLM-use frequency	Ordinal logistic regression	*p* = 0.630	OR = 0.963, 95% CI 0.828–1.12	LLM-use frequency was not associated with integrity concern.
Ordinal model: Integrity concern, Jordan versus UAE	Ordinal logistic regression	*p* < 0.001	OR = 0.299, 95% CI 0.189–0.472	Jordan showed lower odds of higher concern than UAE.
Ordinal model: Integrity concern, Oman versus UAE	Ordinal logistic regression	*p* < 0.001	OR = 0.197, 95% CI 0.106–0.365	Oman showed lower odds of higher concern than UAE.
Correlations: LLM-use frequency and academic-activity breadth	Spearman correlation with BH correction	adjusted *p* < 0.001	*ρ* = 0.289	More frequent use was associated with broader academic use.
Correlations: LLM-use frequency and integrity-safeguard breadth	Spearman correlation with BH correction	adjusted *p* = 0.018	*ρ* = 0.134	A smaller positive association was observed.
Correlations: LLM-use frequency and verification/concern/critical thinking outcomes	Spearman correlation with BH correction	adjusted *p* ≥ 0.128	Not significant	No significant associations after correction.
Country-stratified correlations: Frequency and academic-activity breadth by country	Spearman correlation with BH correction	adjusted *p* ≤ 0.016 in Jordan, UAE, Brazil, Malaysia	*ρ* = 0.284-0.344 in Jordan, UAE, Brazil, Malaysia	Association was present in four countries but not Oman.
Country-stratified correlations: Frequency and verification breadth in Malaysia	Spearman correlation with BH correction	adjusted *p* = 0.009	*ρ* = −0.355	More frequent use was associated with lower verification breadth in Malaysia.

## Discussion

Across five countries, LLM uptake was near-universal and dominated by ChatGPT, with frequent weekly or daily use and broad integration into routine study behaviours. This aligns with the broader pattern that students adopt LLMs primarily for efficiency and immediate academic utility, with discovery and normalisation often happening socially and informally rather than through formal curricula [1,2]. This social uptake framing also aligns with evidence that subjective norms and perceived usefulness are major drivers of intention to use LLM tools, alongside perceived risks [14–16]. In that context, motivations centred on time-saving, conceptual clarification, and summarisation suggests that LLMs are becoming embedded in everyday learning practices, which makes the absence or unevenness of institutional guidance more important [17,18].

These findings are especially relevant to dental education because dentistry requires the integration of declarative knowledge, visual-spatial reasoning, psychomotor skill, and clinical judgement. The finding that 75.3% of students used LLMs to understand complex concepts suggests that these tools are already being used within the cognitive and explanatory layer of dental learning. In this sense, LLMs may help students rehearse procedural sequences, clarify terminology, compare treatment rationales, and prepare for case-based discussion before entering simulation or clinical settings. However, the educational value of this text-mediated support depends on how it is connected to the practical and embodied dimensions of dental training. For example, an LLM may help a student understand the principles of cavity design, adhesive protocols, endodontic access, or treatment planning, but that understanding must still be translated into spatial judgement, tactile control, visual discrimination, and supervisor-calibrated clinical performance. The disciplinary issue, therefore, is how dental curricula can deliberately connect LLM-supported conceptual learning with simulation, preclinical laboratory work, visual case analysis, chairside reasoning, and feedback-rich clinical teaching.

A central contribution of this dataset is that LLM use is a collection of behaviours, spanning low-stakes learning support and higher-stakes, integrity-sensitive uses. Although higher-stakes uses (exam assistance and draughting academic papers) were less common than learning support, their prevalence was non-trivial and varied by cohort. This matters because high-stakes contexts amplify downstream harms, including unfairness in assessment and erosion of trust in qualifications, which dental and medical education stakeholders have explicitly raised as a governance and integrity concern [4,13]. This concern also aligns with a recent work showing that academic dishonesty is not a trivial infraction but a professional formation issue with potential downstream implications for practice and trust [19]. A particularly important pattern is that cross-country variation was evident in higher-stakes uses, where policy clarity and enforcement may be especially important.

Integrity-safeguard findings also warrant a sharper interpretation than ‘most students take at least one step’. The modal safeguard was paraphrasing or rewriting, with sizeable uptake of paraphrasing or humanising tools, citations, and plagiarism-detection checks. This pattern points to a procedural orientation to integrity management, where safeguards appear to centre more on modifying, checking, or legitimising text than on transparent disclosure and evidence traceability. That is, the emphasis is on transforming text or cleaning outputs, rather than on traceability, evidence, and epistemic accountability. This framing aligns with concerns in dental education that LLMs can mislead learners via inaccurate content and fabricated or unreliable citations, making citation-like outputs particularly risky if students are not trained to verify sources and evidence quality [4,13], and with empirical evaluations in evidence-based dentistry reporting that LLM responses may be vague, occasionally inaccurate, potentially outdated, and often lack explicit sourcing, reinforcing the need for critical appraisal rather than surface-level citation handling [20]. It also aligns with a broader concern that procedural workarounds can coexist with, rather than replace, ethical judgement and professional accountability [19]. The low prevalence of disclaimers, and guideline-following was relatively uncommon overall, supports the argument that norms of transparency around AI assistance are still weakly institutionalised in student practice [6–9,12], and also supports educator-level concerns about unclear or inconsistent institutional guidance in dental education [17].

A related issue is the tension between reported verification behaviour and perceptions of LLM reliability across cohorts. Many students reported verifying often or always, yet cohort differences were evident, with Oman showing lower on verification frequency and some traditional cross-checking strategies. This matters because the literature consistently warns that novices and less experienced learners are disproportionately vulnerable to accepting plausible-sounding misinformation and hallucinations, precisely because they lack the domain knowledge and metacognitive routines required for effective risk evaluation of outputs [1,11]. Qualitative evidence from medical students similarly describes LLMs as useful for learning and efficiency while simultaneously raising concerns about erroneous outputs, privacy, misuse, and overreliance, reinforcing that perceived benefit can coexist with recognised risk [21]. Higher exposure to LLMs therefore does not automatically translate into higher scepticism or stronger verification habits, even if frequency modestly trends in the right direction. This is consistent with the broader view that critical thinking in an LLM environment becomes less about generating answers and more about supervising, interrogating, and validating answers, which requires explicit training rather than mere exposure [11,22]. The results suggest that intensity of use is a stronger predictor of what students do with LLMs (breadth of academic activities) than of how sceptical or risk-aware they are, while more frequent users showed a modest shift toward verifying ‘often or more’, higher use did not consistently translate into broader verification strategies or heightened integrity concern. This interpretation is strengthened by evidence that different components of LLM literacy do not uniformly promote verification: in one study, greater process understanding predicted more fact-checking, whereas higher self-efficacy and feature knowledge predicted less fact-checking, consistent with a machine-heuristic or overconfidence pathway [23]. Complementing this, another study suggests that LLM use can co-occur with cognitive offloading tendencies and uneven metacognitive profiles, which plausibly increases vulnerability to superficial acceptance without structured verification routines [24].

The observed differences in perceived critical thinking impact and ‘keeping learning up-to-date’ beliefs are best interpreted as cohort-based ecological variation rather than national differences. In this study, educational ecology refers to the local conditions that shape how students encounter, interpret, and normalise LLM use, including digital access, institutional policy maturity, assessment design, staff messaging, curricular expectations, peer norms, and the hidden curriculum around acceptable academic support. If one cohort perceives greater difficulty learning to use LLMs effectively, invests more time rather than saving time, or is more sceptical that LLMs keep learning current, this may reflect interacting local conditions rather than simple national or cultural differences. In other words, the observed country-based cohort effect in the models likely captures a blend of institutional policy maturity, teaching norms, assessment pressures, student communication channels, and peer-driven practices [1,2,6–9]. This ecological framing is consistent with work noting that adoption is facilitated by convenience and access but can be hindered by geographic access restrictions and digital literacy deficiencies [16], and by work showing that perceived risk can dampen attitudes even when usefulness is high [15], with related qualitative evidence describing simultaneous perceived utility alongside concerns such as dependency, privacy, and weak referencing practices in student LLM workflows [25]. Therefore, the observed cross-country variation should be interpreted as context-sensitive cohort variation rather than evidence of fixed national patterns.

From a disciplinary perspective, these findings extend the medical-versus-dental contrast highlighted in the literature. Medical students are often described as more willing to use LLMs as a general-purpose academic tool, while dental students tend to report higher ethical concern and encounter more technical limitations, especially where image-based reasoning is central [4,10,13,26,27]. This study strengthens that conversation by showing that even in predominantly learning-oriented use, integrity-sensitive behaviours remain present, and the governance gap (uncertainty about guidelines, low transparency practices) is visible in student-reported behaviour. This reinforces the argument that dental education, in particular, cannot rely on informal adoption and must develop context-specific guidance and training, especially given technical constraints and the high premium on evidence-based, precise information [4,13]. Empirical work in evidence-based dentistry also reinforces that limitation-risk profile: LLM outputs may omit sources and include inaccuracies or outdated content, making uncritical uptake particularly problematic in a discipline where evidence traceability and currency are central [20]. Evidence from medical students also shows that even where perceived benefits are prominent, students still articulate risk and overreliance concerns, underscoring that utility does not remove the need for governance and training [21].

The empirical pattern summarised in Table 2 can be organised through an affordance-governance-professional formation lens. At the affordance level, frequent use for concept clarification, summarisation, examination preparation, and assignment-related work reflects the perceived usefulness of LLMs for immediate academic support. At the governance level, differences in guideline awareness, verification practices, and integrity safeguards suggest that student behaviour is shaped by local institutional expectations, assessment conditions, and peer-normalised practices. At the professional formation level, the prominence of paraphrasing, citation, and plagiarism-checking behaviours, together with low disclosure, raises questions about how students learn transparency, evidence traceability, and accountable judgement. This framing connects the descriptive findings to the central educational issue: how dental programmes can help students convert LLM use into disciplined, evidence-accountable practice, while avoiding a narrow focus on procedural text management.

Implications follow directly. First, students need explicit instruction in verification and risk evaluation routines: how to cross-check claims against primary sources, how to treat references as suspect until verified, and how to document AI assistance transparently [11–13]. This should include centralised AI-literacy training that establishes baseline expectations around acceptable use, disclosure, privacy, hallucinated content, fabricated references, and uncertainty management. However, centralised training should be reinforced through embedded dental and clinical instruction, where students practise applying these principles during case-based learning, simulation, preclinical laboratory work, and supervised clinical teaching. In these settings, LLM outputs can be checked against clinical guidelines, primary evidence, case findings, discipline-specific reasoning standards, and supervisor feedback. Second, prompt engineering should be taught as a safety skill rather than a productivity hack, paired with training that prevents overconfidence and supports calibrated scepticism [11,23,28]. Third, assessment design should adapt. If high-stakes use is occurring, institutions must either redesign assessments to resist unauthorised AI assistance or explicitly integrate permitted AI use with enforceable expectations for disclosure, justification, and evidence trails [1,4,6–9]. This aligns with calls for structured learning activities that require critical evaluation and source checking, rather than assuming metacognitive regulation will develop automatically through use, and with evidence that cognitive offloading tendencies can coexist with LLM use [24]. Finally, the policy environment matters. Work analysing institutional AI documents suggests that many institutions prioritise academic honesty and plagiarism language, while leaving gaps in infrastructure, compliance mechanisms, and longer-term strategic integration [6–9]. The uneven awareness of guidelines and low prevalence of transparent disclosure practices support moving from ad hoc messaging to coherent governance that students can actually apply. This aligns with dental education stakeholders’ calls for clearer guidance and staff-student training around chatbots and LLMs [17], and mirrors evidence from clinicians showing substantial LLM uptake alongside scarce workplace guidelines [18]. Given broader concerns about professional integrity in dental training, governance that connects academic conduct to professional identity formation may be particularly important [19].

Several limitations should be acknowledged. A formal a priori sample size calculation was not performed, as the study was exploratory and relied on voluntary participation through participating institutions. The data are self-reported and cross-sectional, so causal effects of LLM use on learning or integrity outcomes cannot be inferred. AI proficiency was also assessed through self-report rather than an objective competency measure, which may limit the interpretability of this variable. Country comparisons are informative but should be interpreted as comparisons between country-based institutional cohorts rather than nationally representative estimates. The study used convenience sampling through academic networks, with uneven sample sizes and potentially different mixes of programme types. Although respondents were recruited from five countries, most countries were represented by one institution, and the UAE by two institutions. Therefore, observed differences may reflect institution-specific policies, teaching cultures, assessment practices, student communication channels, or local exposure to AI guidance, rather than national patterns alone. Wider international sampling across additional regions and multiple institutions per country would be needed to strengthen external validity. Formal response rates could not be calculated because the total number of eligible students who received the survey invitation was not consistently available across all participating institutions. Also, perceived critical thinking impact does not measure actual performance. These limitations point to clear next steps: longitudinal designs, objective verification tasks, and evaluation of targeted training interventions in prompt engineering, verification, and ethical reasoning [13,14,19]. The limitations also motivate evaluation approaches that directly test verification competence under realistic task conditions, given evidence that confidence or tool familiarity may suppress factchecking [23], and that LLM use can align with cognitive offloading tendencies [24].

## Conclusion

In this multi-country survey of senior dental students, LLM use was widespread and largely centred on ChatGPT with frequent integration into routine learning activities. Students primarily used LLMs for efficiency-oriented tasks, including concept clarification, summarisation, and exam preparation, and many perceived an overall academic benefit. However, higher-stakes uses, including exam-related assistance and draughting academic papers, were also reported and varied across countries. Integrity and verification practices were common but uneven, with safeguards often centred on text-processing rather than transparency and evidence-traceability behaviours, and with substantial cross-country differences in verification frequency, guideline awareness and perceived risks. Greater intensity of LLM use was associated with broader engagement across academic activities, but did not consistently translate into stronger scepticism, broader verification strategies or heightened integrity concern. These findings support the need for dental programmes to move beyond ad hoc messaging toward explicit and skills-based training in verification and risk evaluation, paired with clear enforceable institutional guidance and assessment designs that account for real-world patterns of LLM use across contexts.

## Supplementary Material

Supplementary Material SurveyQuestions.docx

## Data Availability

The datasets generated or analysed during this study are available from the corresponding author on reasonable request.
