# Supplementary material for: Large language model use in dental education: a cross-sectional multi-country study
Source: Med Educ Online. 2026 Aug 6;31(1):2707729. doi: 10.1080/10872981.2026.2707729 (PMC13449427; doi:10.1080/10872981.2026.2707729)
Supplement: Supplementary file 1 [file ZMEO_A_2707729_SM3869.docx]

**Questionnaire**

**1. Country**

**2. Age**

**3. Gender**

- Male
- Female

**4. How would you rate your proficiency in using artificial intelligence tools?**

- Beginner
- Intermediate
- Advanced
- Expert

**5. Are you familiar with Large Language Models (LLMs) such as ChatGPT, GPT-4, Bard, Claude, DeepSeek or Med-PaLM?**

- Yes
- No

**6. Which of the following LLMs have you used for academic purposes? (Select all that apply)**

- ChatGPT
- Bard
- Claude
- DeepSeek
- Med-PaLM
- None
- Other (please specify):

**7. How frequently do you use LLMs for academic purposes?**

- Daily (almost every day)
- Several times a week
- Weekly (about once a week)
- Monthly
- Rarely
- Never

**8. Are you aware of any institutional or university guidelines on using AI/LLMs for academic or clinical tasks?**

- Yes
- No
- Not sure

**9. (If Yes) Have these guidelines influenced how you use LLMs?**

- Yes significantly
- Yes somewhat
- No impact

**10. What is your main motivation when using LLMs? (Select all that apply)**

- To save time on studying or completing assignments
- To gain deeper conceptual understanding of complex topics
- To find quick summaries or references
- Curiosity or exploration of new technology
- To overcome language barriers or improve clarity in communication
- To generate new ideas or approaches for projects and research
- To practice question-and-answer formats for exams
- To improve my overall academic performance

**11. In which of the following academic activities have you used LLMs? (Select all applicable)**

- Understanding complex medical/dental concepts
- Exam or quiz preparation (practice questions, study guides, etc.)
- Assignment research (finding and summarizing sources)
- Generating or writing assignments (essays, reports, etc.) including language editing.
- Generating or drafting research papers
- Helping solve problems during examinations or test-taking
- Clarifying lecture content
- None (I have not used LLMs for any academic activities)

**12. In your experience, how has using LLMs impacted time spent for your academic work compared to traditional resources?**

- Significantly less time overall
- Somewhat less time overall
- No change in time spent
- Somewhat more time overall
- Significantly more time overall

**13. In your experience, how has using LLMs impacted Academic Outcomes compared to traditional resources?**

- Significantly improved
- Somewhat improved
- No noticeable change
- Not sure

**14. How would you rate the difficulty of learning to effectively use LLMs for your academic tasks?**

- Very easy (I picked it up immediately with no special effort)
- Somewhat easy (it required minimal practice or exploration)
- Moderate (I needed to spend some time learning and experimenting)
- Somewhat challenging (I invested a fair amount of time or found it tricky)
- Very challenging (I’m still struggling to use LLMs effectively)

**15. LLMs provide more up-to-date information than conventional resources:**

- Strongly agree
- Agree
- Neutral
- Disagree
- Strongly disagree

**16. In your experience, how would you rate the information provided by LLMs compared to textbooks and lectures in terms of medical accuracy, reliability, and currency (how up-to-date)??**

**17. In your experience, how would you rate the information provided by LLMs compared peer-reviewed articles in terms of medical accuracy, reliability, and currency (how up-to-date)??**

**18. In your experience, how would you rate the information provided by LLMs compared online databases & forums, in terms of medical accuracy, reliability, and currency (how up-to-date)??**

**19. In your experience, how would you rate the information provided by LLMs compared to exam preparation manuals in terms of medical accuracy, reliability, and currency (how up-to-date)??**

**20. In your experience, how would you rate the information provided by LLMs compared to question banks in terms of medical accuracy, reliability, and currency (how up-to-date)??**

21. Do you **verify the accuracy of responses provided by LLMs?**

- Always
- Often
- Sometimes
- Rarely
- Never

**22. If you verify LLM outputs, how do you usually do it? (Select all that apply)**

- Compare with textbooks
- Compare with lecture notes
- Look up peer-reviewed articles or guidelines
- Consult with faculty
- Consult with peers
- Use alternative online databases and forums
- Other (please specify):

23. What barriers or difficulties have you encountered when using LLMs?

- Difficulty generating effective prompts
- Encountering misinformation or inaccuracies
- Technical or accessibility issues
- Privacy concerns
- Others, please specify
- None

**24. When using LLM-generated content, plagiarism or academic integrity issues pose significant concerns?**

- Strongly agree
- Agree
- Neutral
- Disagree
- Strongly disagree

**25. Which of the following steps do you typically take to avoid plagiarism or maintain academic integrity when using LLM-generated content? (Select all that apply)**

- Paraphrasing or rewriting the content in my own words
- Using a dedicated paraphrasing or “humanizing” tool (e.g., QuillBot, Wordtune)
- Providing in-text citations or references for any borrowed ideas or data
- Checking my work with plagiarism-detection software (e.g., Turnitin)
- Summarizing the content and citing sources explicitly
- Comparing outputs from multiple AI tools to verify consistency and originality
- Consulting faculty or peers for feedback or review
- Following any institutional or departmental guidelines for AI-generated content
- Indicating (via a disclaimer) that an AI tool contributed to the work
- I do not take any special steps regarding plagiarism or integrity issues

**26. To what extent do you believe relying heavily on LLMs affects critical thinking and independent learning skills?**

- Significantly detrimental (It greatly reduces critical thinking/independent learning)
- Somewhat detrimental
- No noticeable impact
- Somewhat beneficial
- Significantly beneficial (It meaningfully enhances these skills)
